# The occurrence of cystic echinococcosis in slaughtered livestock in Jahrom, south of Iran

**DOI:** 10.1016/j.parepi.2022.e00274

**Published:** 2022-09-08

**Authors:** Manoochehr Shabani, Kavous Solhjoo, Ali Taghipour, Abdolreza Sotoodeh Jahromi, Saina Karami, Belal Armand

**Affiliations:** a Department of Microbiology, Jahrom Branch, Islamic Azad University, Jahrom, Iran; b Zoonoses Research Center, Jahrom University of Medical Sciences, Jahrom, Iran; c Department of Medical Parasitology and Mycology, School of Medicine, Jahrom University of Medical Sciences, Jahrom, Iran; d Department of Parasitology, School of Medicine, Ahvaz Jundishapur University of Medical Sciences, Ahvaz, Iran; e Institute of Precision Medicine, Medical and Life Sciences Faculty, Furtwangen University, Furtwangen, Germany

**Keywords:** *Echinococcus granulosus*, Cystic echinococcosis, Livestock, Iran

## Abstract

Cystic echincoccosis (CE) is a major medical and veterinary concern in the world, especially in Iran. Domestic intermediate hosts are an important reservoir for the disease spread. The purpose of this study was to determine the prevalence of CE in slaughtered livestock in Jahrom, south of Iran. In this cross-sectional study, a total of 3074 animals (2325 (75.63%) goats, 423 (13.76%) sheep, and 326 (10.60%) cattle) were inspected macroscopically for CE. In this regard, a questionnaire about the age, sex, infected organ, number of cysts, and cyst fertility was accomplished for each animal. Moreover, PCR was applied by using the cytochrome *c* oxidase I (COX1) and NADH dehydrogenase subunit 1 (nad1) fragments of parasite mitochondrial genomes on some positive samples. Prevalence of CE in sheep, cattle, and goats was 11.34% (48/423), 11.04% (36/326), and 2.79% (65/2325), respectively. In all livestock, CE was more common in females than males. Moreover, the highest infection rate was observed in the age group of more than 72 months. Considering the fertility of cysts, the ratio of the number of fertile cysts to total cysts in sheep and goats were 83.3% (40/48) and 80% (52/65), respectively. All hydatid cysts were infertile in cattle. With regard to the location of the cyst on internal organs, the most were observed in the lungs and liver. Regarding the intensity of infection, 1–5, 6–9 and ≥ 10 cysts were detected in 78.52% (117/149), 6.71% (10/149) and 10.73% (16/149) of infected livestock, respectively. In all livestock, hydatid cyst with a diameter of 1–5 cm was the most frequent with 71.81% (107/149). All 149 cyst samples were subjected to PCR. Among them, a total of 18 samples (six samples of each animal) were prepared for sequencing. G6 was the most dominant. According to the results of present study and the relatively high prevalence of CE in slaughtered livestock in Jahrom, health policy makers, health authorities, and experts should make effective approach in this regard, and implement careful inspections.

## 1 Introduction

Cystic echinococcosis (CE) is a cosmopolitan zoonotic disease caused by the larval (metacestode) stage of the cestode parasite *Echinococcus granulosus* (Azlaf and Dakkak, 2006; Sarkar et al., 2017). The global distribution of CE is estimated at 1–3.6 million disability-adjusted life years (DALYs) worldwide; most of these cases occur in low- to middle-income countries (Budke et al., 2006; Torgerson et al., 2015). The life cycle of *E. granulosus* involves of canids (definitive hosts) and herbivores/omnivores animals (intermediate hosts) (Gemmell et al., 1986; Torgerson and Heath, 2003). Infection of human and other intermediate hosts (mainly livestock) occurs accidentally by ingestion of infective eggs from soil, water and vegetables which develop into a larval stage in internal organs (Tamarozzi et al., 2020). A cyst of *E. granulosus* comprises of two parasite-derived layers; an inner nucleated germinal layer and an outer acellular laminated layer which is then surrounded by a host-produced fibrous capsule (Beigh et al., 2018). CE mainly affect the liver (50–77%) and lungs (15–47%) of the intermediate hosts (Botezatu et al., 2018; Torabi et al., 2021). However, it is found to a lesser extent in other organs such as the spleen (2–4%) and the kidneys (0.5–8%) (Torabi et al., 2021). Therefore, CE can cause a broad range of different complications.

Although CE causes severe signs/symptoms and possible death if left untreated, it can lead to economic losses from treatment costs, reduction in growth, fecundity and milk production of infected livestock (Budke et al., 2006). At the abattoir, detecting CE during routine meat inspection will lead to condemnation of the infested offal and carcasses (Abdulhameed et al., 2018). Also, fertile cysts in livestock carcasses are very crucial for maintenance of the sheep-dog transmission cycle (Abdulhameed et al., 2018; Torgerson and Heath, 2003). According to studies in Jordan (Torgerson et al., 2001) and Iraq (Abdulhameed et al., 2018), estimated the annual loss of edible liver and lungs due to CE at around US$850,000 and US$72,470 annually, respectively.

Recently, a meta-analysis study in Iran showed that the pooled prevalence of CE among camel, buffalo, cow, sheep, and goats were estimated to be 18.3% (95% CI: 5.5–46.4%), 5.2% (95% CI: 3.5–7.7%), 4.8% (95% CI: 3.5–6.5%), 4.3% (95% CI: 3.2–5.8%), and 3.7% (95% CI: 2.6–5.2%), respectively (Vaisi-Raygani et al., 2021). Therefore, understanding the regional epidemiology of CE in intermediate hosts is critical in order to plan for control and prevention strategies. In the present study, the survey area is located in Jahrom city (South of Iran) where livestock and dogs are frequently together. Finally, this study aimed at investigation of the prevalence of CE in cattle, sheep, and goats slaughtered for food consumption in Jahrom city from Fars province, Iran.

## 2 Materials and methods

### 2.1 Study area

The present study was performed from January 2017 to June 2017 in Jahrom city, which is located in south of the Fars province. Regarding the geographical coordinates, this city is located between Latitude: N 28° 31′ 3.8784“ and Longitude: E 53° 34’ 27.7932”. Jahrom has a hot semi-arid climate, the average rainfall is approximately 285 mm per year, and the average temperature is about 20 °C. Moreover, the average height of Jahrom is about 1050 m above sea level.

### 2.2 Sample collection

This cross-sectional study was carried out on 3074 animals (2325 (75.63%) goats, 423 (13.76%) sheep, and 326 (10.60%) cattle) at Jahrom slaughterhouses. Then, a questionnaire about the type of animal, age, sex, infected organ and number of cysts was accomplished for each animal. The number of cysts was counted to determine the intensity of the infection. In this regard, intensity of infection was divided into three categories: light infection (1–5 cysts), moderate infection (6–9 cysts), and intense infection (≥10 cysts). The internal organs infected with hydatid cyst were collected from the slaughterhouse and transferred on ice to the Parasitology Laboratory of Zoonosis Research Center, Jahrom University of Medical Sciences for identification using the description of Soulsby (Soulsby, 1982).

### 2.3 Determination of cyst fertility

All cysts were examined for degeneration and calcification. Then, non-calcified hydatid cysts selected for fertility study. The cyst wall was incised with sterile scalpel blade and the content was transferred into a sterile container. The content was surveyed under a microscope (40 x) for the presence of protoscoleces (PSCs). The cysts which contained PSCs were considered as indication of fertility while the absent of PSCs was considered as infertile cysts (Taghipour et al., 2021). Considering molecular analysis, hydatid fluid and PSCs were, also, preserved in 70% ethanol.

### 2.4 DNA extraction and polymerase chain reaction (PCR)

The genomic DNA of 50 μl of fertile PSCs was extracted by manual method, using phenol chloroform (Barazesh et al., 2018). Purified DNA samples were stored at −20 °C until further use. In the next step, cytochrome *c* oxidase I (COX1) and NADH dehydrogenase subunit 1 (nad1) fragments of parasite mitochondrial genomes were targeted and PCR-amplified using primers JB3 (F): (5′-TTTTTT GGGCATCCTGAGGTTTAT-3′) and JB4.5 (R): (5’-TAAAGAAAGAACATAATG AAAATG-3′) for cox1 (Barazesh et al., 2018; Bowles et al., 1992) and eg-nad1 (F): (5’-AGGTTTGCCGATTTGTTGAAG-3′) and eg-nad1 (R): (5’-CAACAGCATAAAGCGCAAAAAATAAC-3′) for nad1 (Busi et al., 2007). Then, PCR products were visualized by electrophoresis on 1.5% agarose gel stained with SYBR Safe. Finally, PCR products of some positive samples were sequenced using Applied Biosystems 3730/3730xl DNA Analyzers (Bioneer, South Korea) and the results were compared using BLAST software in the GenBank database.

## 3 Results

### 3.1 Parasitology survey

The overall prevalence of CE was determined to be 4.84% (149/3074) in livestock. In this regard, the prevalence of CE was found in 11.34% (48/423) in sheep,11.04% (36/326) in cattle, and 2.79% (65/2325) in goats. CE was more common in females than males in all livestock (Table 1). Moreover, the highest infection rates were observed in the age group of more than 72 months (Table 1). With regard to the fertility of cysts, the ratio of the number of fertile cysts to total cysts in sheep and goats were 83.3% (40/48) and 80% (52/65), respectively. All hydatid cysts were infertile in cattle. Considering the location of the cyst on internal organs, the most were observed in the lungs and liver (Table 2). Also, the frequency of CE according to the location of cyst formation in each animal is shown in Table 2. Regarding the intensity of infection, 1–5, 6–9 and ≥ 10 cysts were observed in 78.52% (117/149), 6.71% (10/149) and 10.73% (16/149) of infected livestock, respectively. Furthermore, the frequency of CE according to the intensity of infection in each animal is shown in Table 3. In all livestock, hydatid cyst with a diameter of 1–5 cm was the most frequent with 71.81% (107/149). The frequency of CE is given in Table 4 according to the size of the cyst diameter in each livestock.

**Table 1:** Age and sex associated with CE among livestock slaughtered for food consumption in Jahrom city from Fars province, Iran.

Variables	0–24 months		24–48 months		48–72 months		72 < months		Male		Female	
	Examined (n)	Positive n (%)	Examined (n)	Positive n (%)	Examined (n)	Positive n (%)	Examined (n)	Positive n (%)	Examined (n)	Positive n (%)	Examined (n)	Positive n (%)
Cattle	42	4 (9.52)	59	4 (6.77)	129	14 (10.85)	96	14 (14.58)	195	20 (10.25)	131	16 (12.21)
Sheep	68	10 (14.70)	96	7 (7.29)	123	10 (8.13)	136	21 (15.44)	253	26 (10.27)	170	22 (12.94)
Goat	598	10 (1.67)	514	19 (3.69)	691	14 (2.02)	522	22 (4.21)	1395	37 (2.65)	930	28 (3.01)

**Table 2:** The frequency of CE according to the location of cyst formation in each animal.

Internal organ	Lung	Liver	Kidney	Femur	Liver+lung	Other organ
Cattle	17	4	2	0	12	1
Sheep	27	13	0	1	4	0
Goat	48	8	0	3	4	2
Total	92	25	2	4	20	3

**Table 3:** The frequency of CE according to the intensity of infection in each animal.

Number of cyst	Light infection (1–5 cysts)	Moderate infection (6–9 cysts)	Intense infection (≥10 cysts)
Cattle	29	1	4
Sheep	35	4	6
Goat	53	5	6
Total	117	10	16

**Table 4:** The frequency of CE according to the size of the cyst diameter in each livestock.

Cyst size	1 cm>	1–5 cm	6–10 cm	10 cm<
Cattle	2	29	2	1
Sheep	14	31	0	0
Goat	14	47	3	0
Total	3	107	5	1

### 3.2 Molecular survey

**Fig. 1:**
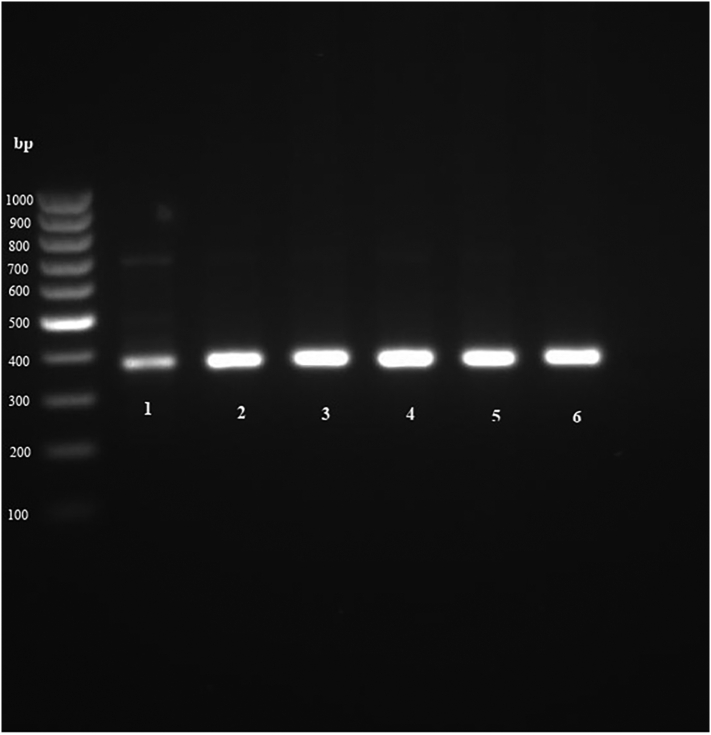
PCR-product of *Echinococcus granulosus* based on band size by COX 1 gene.

**Fig. 2:**
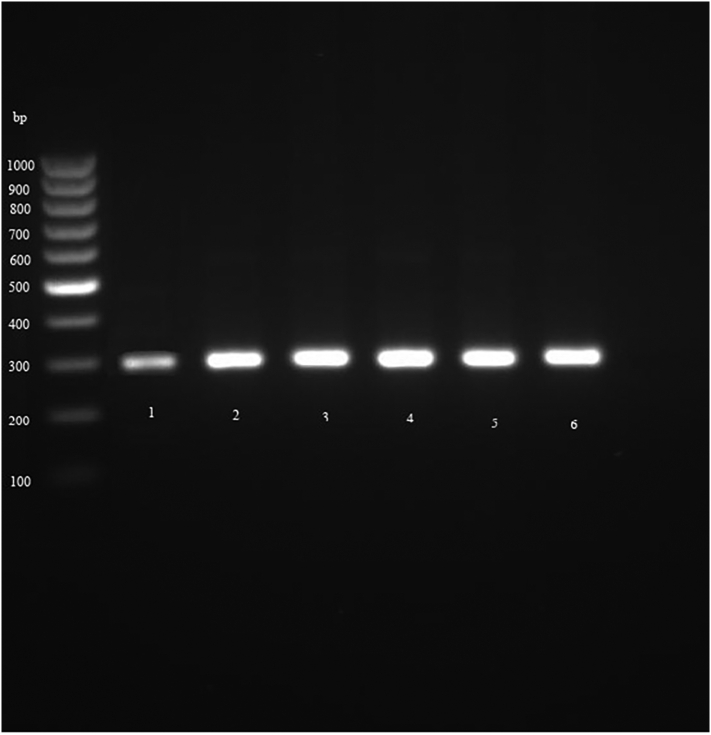
PCR-product of *Echinococcus granulosus* based on band size by nad1 gene.

All 149 cyst samples were subjected to molecular analysis and bands were observed for all of them. Then, a total of 18 samples (six samples of each animal) were prepared for sequencing (nine samples for COX1 and nine samples for nad1). Among them, 16 samples were sequenced and sequencing failed in two samples. Also, animal type, genotypes and accession numbers for each gene (COX1 and nad1) are shown in Table 5. Among the genotypes, G6 was the most dominant (Table 5). It should be noted that both COX1 and nad1 genes showed the same genotypes through the BLAST in the GenBank database (Table 5). Some PCR-products of the COX1 and nad1 genes are shown in Fig. 1, Fig. 2, respectively.

**Table 5:** Accession numbers and genotype assignment of hydatid cysts by sequencing analysis of two mitochondrial genes (COX1 and nad1) studied according to their host in Jahrom.

Isolate	Host	AC. No. (COX1)	AC. No. (nad1)	Genotype
ZRCJ1	Cattle	JN545835	JX067637	G8
ZRCJ2	Cattle	JX067639	KU587711	G3
ZRCJ3	Cattle	JX067640	JX067638	G8
ZRCJ4	Sheep	KU220240	KU601745	G6
ZRCJ5	Sheep	KU220241	KU495927	G6
ZRCJ7	Goat	KU359037	KU641482	G6
ZRCJ8	Goat	KU359038	KU681089	G6
ZRCJ9	Goat	KU376088	KU681088	G6

## 4 Discussion

The results of present study show that the prevalence of CE in slaughtered livestock in Jahrom is 4.84%. In general, the prevalence of CE in livestock is heterogeneous in different cities from Iran. The prevalence of this study is almost consistent with the reports of previous studies in livestock in Shahrekurd (6%) (Sabbaghian et al., 1975), Shiraz (5.4%) (Mehrabani et al., 1999), and Ahwaz (4.6%) (Ahmadi and Meshkehkar, 2011) of Iran. However, the reported rate is much lower than the results from Ardebil (60.8%) (Daryani et al., 2007) and Mazandaran (54.1%) (Ziaei et al., 2011). These differences may be due to the knowledge of health inspectors, poor facilities for carcass inspection in slaughterhouses, regional differences and research methods (Randolph et al., 2007). In the present study, the prevalence of CE was as 11.34%, 11.04%, and 2.79%, in sheep, cattle, and goats, respectively. Although animal husbandry plays a major role in human nutrition and socioeconomic development, zoonotic diseases such as CE in livestock may impose costs on the economies of countries (Nayar, 1974; Sarıözkan and Yalçın, 2009). In one research at a national level, financial losses due to CE of cattle, sheep, and goats were estimated as 32, 54.1, and 2.7 million dollars, respectively (Razi Jalali et al., 2005; Vaisi-Raygani et al., 2021). This loss for the removal of carcasses and internal organs of livestock infected with CE was calculated as high as 1 billion dollars in Saudi Arabia over a three-year period (Ibrahim, 2010; Vaisi-Raygani et al., 2021). Hence, several factors including long-term planning, proper financing by the government, raising public awareness of hydatidosis, daily visits to meat sources, prevention of stray dogs around slaughterhouses, proper vaccination (such as the EG95 for sheep) to control and prevent hydatidosis are essential (Moazeni, 2008).

In this study, the prevalence of CE in lungs was higher than that in liver in all livestock. In this regard, many studies have shown the prevalence of CE in lungs or liver of livestock (Azami et al., 2013; Elsami et al., 1981). As a result, the lungs were condemned more than the liver because of the high affinity of the parasite to infect lungs and due to a lower price.

In our study, the prevalence of CE was higher in older livestock than in younger livestock. It should be noted that the prevalence of CE in younger sheep (0–24 months) was similar to the prevalence in older sheep, which for a deeper understanding of this issue, a larger sample size of animals should be examined. Although young livestock are often slaughtered for meat consumption because their meat is lighter in color and cooks faster, the prevalence of CE is higher in older livestock (Torgerson and Heath, 2003). One reason for this higher prevalence may be that older livestock are more likely to be exposed to sources of infection than younger livestock (Qingling et al., 2014). Regarding the livestock age at the time of slaughter can also be considered as a key factor in reducing the rate of infection spread.

In the present study, the viability of PSCs of fertile cysts for sheep and goats were about 83.3% and 80%, respectively. Cysts may have different fertility rates depending on the geographical location, type of infected host, cyst location, size, and type of cyst. Status of fertile cysts in various livestock provide reliable indicators of the importance of each type of livestock as a potential source of infection to dogs.

Up to now, ten genotypes of *E. granulosus* (G1–10) have been characterized, including *E. granulosus sensu stricto* (G1–3), *E. equinus* (G4), *E. ortleppi* (G5), and *E. canadensis* (G6–10) (Amer et al., 2015). The results of our study showed that G6 was more abundant than other genotypes. A systematic review in Iran has shown that G6 is the most abundant genotype after G1 (Khademvatan et al., 2019). Although three genotypes G6 (five cases), G8 (two cases), and G3 (one cases) were reported in the present study, it should be noted that due to limited financial resources, a small number of samples were sequenced. Therefore, in order to have a deeper understanding of the epidemiological status of genotypes, it is necessary to sequence more samples.

In summary, the prevalence of CE is higher in cattle and sheep than in goats in the study area. This data is of significance largely from the zoonotic point of view and the role livestock play as being a major source of meat in this part of the country. Therefore, effort should be made to control the transmission of cysts from slaughter houses by the safe disposal of infected offal and carcass.

## Authors' contribution

All authors contributed to study design. KS and MS contributed to all parts of the study. ASJ and BA contributed to study implementation. AT, SK, and MS collaborated in the analysis and interpretation of data. AT and KS collaborated in the manuscript writing and revision. All the authors commented on the drafts of the manuscript and approved the final version of the article.

## Funding

This study was supported by Zoonosis Research Center of Jahrom University of Medical Sciences, Iran, grant was awarded to Kavous Solhjoo.

## Availability of data and materials

All data during study are included in this manuscript.

## Ethics approval and consent to participate

This study was approved by Jahrom University of Medical Sciences Ethics Committee.

## Consent for publication

Not applicable.

## Declaration of Competing Interest

The authors declare that there is no conflict of interest regarding the publication of this article.
